# Blood pressure in young adulthood and residential greenness in the early-life environment of twins

**DOI:** 10.1186/s12940-017-0266-9

**Published:** 2017-06-05

**Authors:** Esmée M Bijnens, Tim S Nawrot, Ruth JF Loos, Marij Gielen, Robert Vlietinck, Catherine Derom, Maurice P Zeegers

**Affiliations:** 1 Centre for Environmental Sciences, Hasselt University, Agoralaan Building D, 3590 Diepenbeek, Belgium; 2 Department of Complex Genetics, NUTRIM School of Nutrition and Translational Research in Metabolism, Maastricht University Medical Centre, P.O. Box 616, 6200 MD, Maastricht, The Netherlands; 3 Department of Public Health, Leuven University (KU Leuven), Kapucijnenvoer 35, 3000 Leuven, Belgium; 4 The Genetics of Obesity and Related Metabolic Traits Program, The Charles Bronfman Institute for Personalized Medicine, The Mindich Child Health and Development Institute, The Icahn School of Medicine at Mount Sinai, 1468 Madison Ave, New York, NY 10029 USA; 5 Centre of Human Genetics, University Hospitals Leuven, Herestraat 49, 3000 Leuven, Belgium; 6 Department of Obstetrics and Gynaecology, Ghent University Hospital, De Pintelaan 185, Ghent, Belgium; 7 CAPHRI School for Public Health and Primary Care, Maastricht University, Maastricht, The Netherlands

**Keywords:** Blood pressure, Greenness, Early-life environment

## Abstract

**Background:**

Previous research shows that, besides risk factors in adult life, the early-life environment can influence blood pressure and hypertension in adults. However, the effects of residential traffic exposure and residential greenness in the early-life on blood pressure in young adulthood are currently unknown.

**Methods:**

Ambulatory (24-h) blood pressures of 278 twins (132 pairs) of the East Flanders Prospective Twins Study were obtained at the age of 18 to 25 years. Prenatal and adulthood residential addresses were geocoded and used to assign prenatal and postnatal traffic and greenness indicators. Mixed modelling was performed to investigate blood pressure in association with greenness while adjusting for potential confounding factors.

**Results:**

Night-time systolic blood pressure was inversely associated with greenness at the residential address in twins living at the same address their entire life (non-movers, *n* = 97, 34.9%). An interquartile increase in residential greenness exposure (1000 m radius) was associated with a 3.59 mmHg (95% CI: -6.0 to −1.23; *p* = 0.005) lower adult night systolic blood pressure. Among twins who were living at a different address than their birth address at time of the measurement (*n* = 181, 65.1%), night-time blood pressure was inversely associated with residential surrounding greenness at adult age as well as with residential greenness in early-life. However after additional adjustment for residential greenness exposure in adulthood, only residential greenness exposure in early-life was significantly associated with night systolic blood pressure. While no significant effect of adult residential greenness with adult blood pressure was observed, while accounting for the early-life greenness exposure.

**Conclusions:**

Lower residential greenness in the early-life environment was independently associated with a higher adult blood pressure. This indicates that residential greenness has persistent effects on blood pressure.

**Electronic supplementary material:**

The online version of this article (doi:10.1186/s12940-017-0266-9) contains supplementary material, which is available to authorized users.

## Background

High blood pressure is the leading global risk factor for cardiovascular disease and mortality in the world [1, 2]. Long-term exposure to air pollution has been associated with increased blood pressure [3] and measures of atherosclerosis, including carotid-intima-media thickness [4]. In a meta-analysis of 113,926 participants of the ESCAPE study, a weak positive association was found between high traffic exposure in 100 m of the residence and increased systolic and diastolic blood pressure, and elevated risk of prevalent hypertension [5]. Blood pressure tracks over time and childhood blood pressure is closely related with adult blood pressure, increasing later cardiovascular risk [6].

Given that an unfavorable intrauterine environment may contribute to increased blood pressure [7], we hypothesized that exposure to traffic related air pollution and residential greenness during early-life might be associated with higher blood pressure in early adulthood. Prenatal exposure to air pollutants may influence epigenetic changes in placental tissue, which might result in fetal growth disturbance, and subsequently make children more susceptible to the development of cardiovascular pathologies and disease later in life. Until now, studies on residential greenness and land cover on blood pressure are limited. In the few studies conducted in healthy populations of children or young adults, childhood or recent exposures to greenness has been associated with blood pressure [8–10]. A study in 2076 German children showed that lower residential greenness was associated with higher blood pressure in 10 year-old children living in an urbanised area [8]. A Dutch study investigating the effect of neighborhood-level environmental stressors on blood pressure among different ethnic groups observed that living in a neighborhood with a high quality of green space was associated with a lower systolic blood pressure and lower odds of hypertension in Moroccans [9]. Similar but non-significant associations were observed among Dutch and Turkish ethnic groups [9]. In addition, among 3416 pregnant women of the city Kaunas, Lithuania an association was observed between proximity of the place of residence to green spaces and lower blood pressure [10]. To understand the role of early-life traffic related exposure and residential greenness in blood pressure tracking into adulthood, we investigated the association between early-life exposure of these environmental exposures on blood pressure in young adulthood.

## Methods

### Subjects

The East Flanders Prospective Twin Study (EFPTS), a population based register of multiple births in the province of East-Flanders (Belgium), started in 1964 to enrol twins at birth [11]. The twin cohort in our analysis was based on a previously designed twin study containing 424 twin pairs [12]. From this population we could obtain 333 twins born between 1975 and 1982. We excluded twins born before 1975 since no major changes in the road network have occurred in East-Flanders since 1974. Their addresses at birth and at adult age were geocoded. Twins with missing data were excluded; gestational age (*n* = 4), parental education level (*n* = 17), BMI (*n* = 1), 24 h urinary sodium and potassium (*n* = 6), Gamma-Glutamyl Transferase (*n* = 9), age mother (*n* = 9), neigbourhood SES 1994 (*n* = 3), blood pressure (*n* = 6), resulting in a final study population of 278 persons, 170 monozygotic and 108 dizygotic twins. Informed consent was obtained and ethical approval was given by the Ethics Committee of the Faculty of Medicine of the Katholieke Universiteit Leuven.

### Collection maternal and neonatal data

Data recorded by the obstetrician at birth included gestational age, birth weight, sex of the twins and parental ages. Gestational age was based on the mother’s last menstruation and was calculated as the number of completed weeks of pregnancy. Zygosity was determined by sequential analysis based on sex, choriontype, blood groups based on umbilical cord blood, placental alkaline phosphatase, and, since 1982, DNA fingerprints [13]. After DNA-fingerprinting, zygosity was determined with a 99.9% probability. At a later stage, the parents of the twins filled out questionnaires. In this way, maternal smoking during pregnancy and parental education were collected retrospectively. Educational level as a proxy of socio economic status (SES) was categorized into three groups according to the Belgian education system; no education or primary school, lower secondary education, and higher secondary education and tertiary education. In addition to individual SES data, we gathered information on neighborhood SES. Based on their home address, all mothers were assigned to statistical sectors (average area = 1.55 km^2^), the smallest administrative entity for which statistical data are produced by the Belgian National Institute of Statistics (NIS). Belgian census data (FOD Economie/DG Statistiek) derived from the NIS were used to define neighborhood SES based on annual household income in the year 1994.

### Collection data adulthood

Biometric and laboratory measurements were obtained at the research centre during a 2 h morning session. Standing height and weight were measured as described in detail before [14]. BMI was calculated as body mass (in kg) divided by squared height (in m). The twins completed questionnaires to obtain information on smoking status and physical activity. Regarding habitual physical activity, the twins rated themselves on a 10-point scale after brief instructions, 1 represented very little and 10 very intensive physical activity.

Fasting blood samples were drawn; Gama-Glutamyl Transferase was measured on an Olympus AU600 Auto-Analyzer (Kyoto, Japan). Potassium and sodium were determined in 24 h urine with ion-selective electrodes (Olympus AU600 Auto-Analyzer). Before urine collection, participants were asked to select a day of normal routine, to drink normally and refrain from caffeine use, and to refrain from heavy exercise.

### Ambulatory blood pressure

Ambulatory blood pressure was monitored using the SpaceLabs 90,207 device (SpaceLabs, Inc), as previously described [15]. The ambulatory blood pressure measurements were performed between February 1997 and April 2000. The monitor was applied at home on the nondominant arm. They were instructed to perform normal activity, but not to engage in vigorous physical exercise or contact sports. Recording began between 6.00 and 9.00 AM and was finished 24 h later. The recorders were taken every 15 min during daytime (8.00 AM to 10.00 PM) and every 30 min during night-time (10.00 PM to 8.00 AM). Whenever a reading could not be successfully completed, the measurement was repeated 2 min later. Readings were automatically rejected when systolic blood pressure was >220 mmHg or <70 mmHg and diastolic BP was >140 mmHg or <40 mmHg. In addition to the automatic exclusion of readings by the monitor, individuals were excluded from further analysis when there were no valid measurements in any 2-h period. Night and day were defined with short fixed-clock time periods that ranged from midnight to 6.00 and from 10.00 AM to 8.00 PM. We investigated systolic and diastolic blood pressure during both night and day. Measurements during the morning (6.00 AM to 10.00 AM) and during the evening (8.00 PM to midnight) were excluded. Both periods may be subjected to additional variation due to different activity patterns between individuals.

### Traffic exposure and land use data

Residential addresses of the mothers at time of birth of the twin and the residential addresses of the twin at time of the measurement were geocoded. Distance to the nearest major road, a proxy for traffic-related exposure, was determined using Geographic Information System (GIS) functions. All GIS analyses were carried out using ArcGIS 10 software.

Semi-natural-, forested -, and agricultural areas (greenness), residential and industrial areas in a 5000 m radius from the residential address were estimated based on CORINE Land Cover 2000 (European Environment Agency). This was repeated for 4000, 3000, 2000, 1000, 500, 300 and 100 m buffers. CORINE, the acronym for ‘coordination of information of information on the environment’, was initiated by the European Union and has been taken over by the European Environment Agency. The land cover data is based on satellite data and is dived in 44 classes. It is presented as a cartographic product, at a scale of 1:1,000,000. This database is available for most areas of Europe.

### Assessment of traffic noise

A GIS-based noise model including the Flemish street and railway networks was used to estimate traffic noise levels at the residential address of the mother at time of birth in 5 dB(A)-intervals according to the European Noise Directive (2002/49/EC) (The European Parliament and The Council of The European Union, 2016). The modelling of road noise level included road traffic intensity, vehicle-type-specific traffic density, type of street surface, small-scale topography of the area, and the presence or dimensions of buildings and reflecting objects. Railway noise modelling included the amount of passing trains, type of trains, speed, small-scale topography of the area, and the presence or dimensions of buildings and reflecting objects. Weighted equivalent noise levels in dB(A) for traffic over day-time (based on the weighted yearly average noise level between 7 a.m. to 7 p.m., and 7 p.m. to 11 p.m.,) and at night (yearly average noise level between 11 p.m. and 7 a.m.) were modelled. Exposure to traffic noise was categorized as ≤55 dB, >55 to ≤60 dB, and >60 dB.

### Statistical analysis

For data management and statistical analyses, we used SAS software, version 9.3 (SAS Institute, Cary, NC). All reported *p*-values are two-sided and were considered statistically significant when *p* < 0.05.

Mixed modeling was performed to investigate blood pressure in association with distance to major road and land-use indicators. The twins were analyzed as individuals in a multilevel regression analysis to account for relatedness between twin members by adding a random intercept to the model. The variance-covariance structure was allowed to differ between the three zygosity-chorionicity groups including dizygotic dichorionic, monozygotic dichorionic, and monozygotic monochorionic. Covariates were selected a priori including sex, gestational age, birth weight, birth year, zygosity–chorionicity group, maternal age, age, smoking, physical activity, BMI, 24 h sodium and potassium, gamma-glutamyl transferase, indicators of socioeconomic status (maternal education and neighborhood household income), smoking during pregnancy and noise exposure during the night. To distinguish between exposure early and late in life, we divided the twins in two groups; twins who were living at the same address their whole life (non-movers) and twins who were living at a different address than their birth address at time of the measurement (movers). In the twins who moved during life, we additionally adjusted for land use/distance to major road in adulthood/early-life. Correlation coefficients between greenness in adulthood and early-life are shown in Additional file 1: Table S1. Variance Inflation Factors (VIF) were less than 5, this suggest that the potential issue of multi-collinearity is rather limited (Additional file 1: Table S2).

In addition, we tested potential effect-modifications of the association between land-use indicators and blood pressure by zygosity–chorionicity group.

## Results

The average age of mothers was 27.4 years at the time twins were born and 14.4% of the mothers smoked during pregnancy (Table 1). At time the blood pressure measurements were taken, the twins had a mean (SD) age of 21 (1.9) years, 31% of the twins were current smokers and 5 subjects (1.8%) used antihypertensive medication. The average 24 h urine sodium and potassium excretion was respectively 128.1 ± 59.4 mmol/day and 63.2 ± 28.4 mmol/day and the fasting blood concentration of gamma-glutamyl transferase was 16.6 ± 8.54 U/L. In our final analysis, 95% (*n* = 264) of the participants included both twins from each twin pair, whereas the remaining 5% (*n* = 14) only had one participating twin from each twin pair. A total of 181 (65.1%) of the twins moved between birth address and young adulthood (Table 2). No significant interaction by zygosity-chorionicity (*p* ≥ 0.81) was observed in the model of land-use indicators and blood pressure.

**Table 1:** Study population characteristics

Characteristic			
Maternal	(*n* = 146)	Adulthood	(*n* = 278)
Maternal age, years	27.4 ± 4.5	Age, years	20.9 ± 1.9
Socioeconomic status: maternal education		Body mass index, kg/m^2^	21.1 ± 2.8
Low	59 (40.4)	Smokers, n	87 (31.3)
Middle	32 (21.9)	Physical activity score	4.73 ± 2.2
High	55 (37.7)	Potassium excretion, mmol/day	63.2 ± 28.4
Neighborhood income, euro	19,447 ± 4202	Sodium excretion, mmol/day	128.1 ± 59.4
Smoking during pregnancy	21 (14.4)	Gamma-Glutamyl Transferase, U/L	16.6 ± 8.54
		Zygosity - Chorionicity	
Birth	(*n* = 278)	Dizygotic-Dichorial	108 (38.8)
Gestational age, weeks	37.0 ± 2.4	Monozygotic-Dichorial	83 (29.9)
Neonate birth weight, g	2524 ± 497	Monozygotic-Monochorial	87 (31.3)
Sex		Complete-pair in final study	
Male	149 (53.6)	One twin	14 (5.0)
Female	129 (46.4)	Both twins	264 (95.0)
Twin birth year	1978 ± 2.0	Moved since birth	181 (65.1)

Data presented are means ± standard deviation or number (percentage)

**Table 2:** Distribution of the traffic and land use indicators

	Residential address at birth						Residential address at adult age					
	Geometric mean	Percentile					Geometric mean	Percentile				
		5th	25th	50th	75th	95th		5th	25th	50th	75th	95th
Distance to major road, m	234	23	98	266	591	1881	300	31	134	334	786	1940
Greenness, %:												
5000 m buffer	58	22	54	70	74	83	62	25	59	71	75	85
1000 m buffer	36	0	21	44	67	85	62	7	29	55	70	88
300 m buffer	17	0	0	19	43	83	41	0	2	25	54	94
Residential area, %:												
5000 m buffer	28	13	22	27	37	58	27	13	20	26	35	57
1000 m buffer	44	14	33	51	69	89	38	12	28	43	63	85
300 m buffer	63	18	51	78	98	100	60	3	46	68	87	100
Industrial area, %:												
5000 m buffer	2.80	0.00	1.08	2.90	6.33	9.01	2.48	0.00	0.79	2.67	5.41	9.08
1000 m buffer	7.72	0.00	0.00	0.00	5.52	19.6	7.14	0.00	0.00	0.00	2.43	18.47
300 m buffer, %	12.88	0.00	0.00	0.00	70.00	18.8	11.18	0.00	0.00	0.00	0.00	11.55

**Fig. 1:**
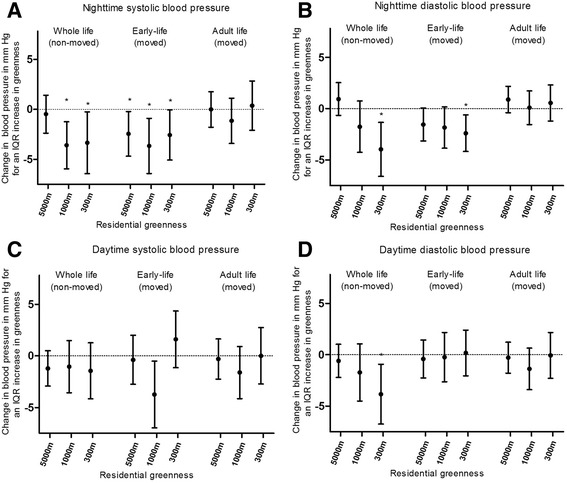
Ambulatory blood pressure (mm Hg) in association with residential greenness exposure. **a** Night systolic, **b** Night diastolic, **c** Day systolic and **d** Day diastolic blood pressure. Adjusted for sex, gestational age, birth weight, birth year, zygosity–chorionicity group, maternal age, age, smoking, physical activity, BMI, 24 h sodium and potassium, gamma-glutamyl transferase, indicators of socioeconomic status (maternal education and neighborhood household income), smoking during pregnancy and noise exposure during the night. In the twins who moved during life, we additionally adjust for greenness exposure in adulthood/early-life. Vertical lines denote 95% confidence intervals. *indicates significant (*p* < 0.05) change in blood pressure for an IQR increase in land use indicators

In 97 twins living at the same address their entire life (non-movers), significant associations were observed between systolic blood pressure during the night and all land use indicators in a 1000 m buffer after adjustment for previously mentioned covariates (Fig. 1 and Additional file 1: Figure S2 and Additional file 1: Figure S3). An interquartile increase in residential greenness exposure within 1000 m residential radius was associated with a decrease of 3.59 mmHg (95% CI: -6.0 to −1.23; *p* = 0.005) in night-time systolic blood pressure. Diastolic blood pressure during night and day was also inversely associated with greenness in a 300 m radius (−4.0 mmHg for IQR increase; 95% CI: -6.6 to −1.3; *p* = 0.006, −3.8; 95% CI: -6.8 to −0.9; *p* = 0.01).

**Fig. 2:**
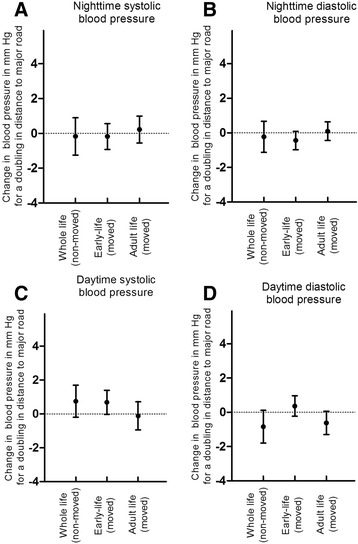
Ambulatory blood pressure (mm Hg) in association with distance to major road**. a** Night systolic, **b** Night diastolic, **c** Day systolic and **d** Day diastolic blood pressure. Adjusted for sex, gestational age, birth weight, birth year, zygosity–chorionicity group, maternal age, age, smoking, physical activity, BMI, 24 h sodium and potassium, gamma-glutamyl transferase, indicators of socioeconomic status (maternal education and neighborhood household income), smoking during pregnancy and noise exposure during the night. In the twins who moved during life, we additionally adjust for distance to major road in adulthood/early-life. Vertical lines denote 95% confidence intervals. *indicates significant (*p* < 0.05) change in blood pressure for a doubling in distance

In the twins who had moved since birth, an increased systolic blood pressure during night was significantly associated with lower greenness and higher residential area in early-life after adjustment for previously mentioned covariates (Additional file 1: Table S1). Exposure to land use indicators in early-life remained significant associated with systolic blood pressure during the night, even after additional adjustment for land use indicators in adulthood (Fig. 1, Additional file 1: Figure S2 and Additional file 1: Figure S3). The association with residential greenness was significant for all buffer sizes (residential greenness radius between 300 m and 5000 m, Additional file 1: Table S3). An interquartile increase in residential greenness exposure in early-life (5000 m radius) was associated with a − 2.47 mmHg (95 CI: -4.7 to −0.2%; *p* = 0.04) decrease in adult night systolic blood pressure, after additional adjustment for residential greenness exposure in adulthood. Diastolic blood pressure during the night was significantly associated with residential greenness in a 300 m buffer (−2.4 mmHg for IQR increase; 95% CI: -4.2 to −0.6; *p* = 0.01; Fig. 1). No associations were observed between blood pressure and distance to major roads in early-life (Fig. 2). Residential greenness indicators were robust for mutually adjustment for distance to major roads, which is a proxy for traffic related exposures.

In the movers, night-time systolic blood pressure was inversely associated with residential surrounding greenness at adult age when no adjustments for early-life exposure were made (Additional file 1: Figure S1). We found no significant effect of adult land use indicators with adult blood pressure, while accounting for the early-life land use indicators (Fig. 1). Only a significant and positive association between diastolic blood pressure during the night and industrial area in a 5000 m buffer remains (−2.4; 95% CI: -4.5 to −0.2; *p* = 0.03; Additional file 1: Figure S3).

## Discussion

The key finding of our paper is that in this twin population, increased early-life exposure to residential greenness was associated with lower blood pressure in young adulthood. These results lend further evidence in support of the developmental origins of disease hypothesis for blood pressure [16] and the role of the residential landscape on the blood pressure life trajectory. Changes in blood pressure in association with land-use exposure were noted during the night and not during the day. Due to physical and mental activity, blood pressure is more variable during the day than during the night. Blood pressure during the night is more related to basal blood pressure and is a better predictor of mortality and coronary heart disease than day-time blood pressure [17].

Our findings are in line with previous studies, showing an association between high residential greenness and low blood pressure in children and adults [8, 9]. However, no studies are available on greenness exposure during pregnancy in association with blood pressure in the newborn. Although, it is reasonable to expect that maternal exposure to greenness also affects newborn blood pressure since a US cohort study of 1059 mothers and their newborn infants shows that maternal systolic blood pressure during the third trimester of pregnancy is associated with the systolic blood pressure among newborns [18].

Residential green space might act via three routes. First, we postulate that the underlying mechanism between surrounding greenness in early-life and blood pressure in young adulthood could be stress. Higher levels of green space in residential neighborhoods, are linked with lower perceived stress and a healthier cortisol levels in women in a deprived urban population [19]. Moreover, the presence of multiple psychosocial stressors in women during pregnancy are associated with 1.5 mmHg higher systolic and diastolic blood pressure in the offspring at the age of 5–7 years [20]. In addition, the prenatal maternal psychological status also does influence blood pressure response to stress in the child when aged 7–9 years [21]. Meta-analysis including 50 cohorts and consisting of 617 data points for systolic blood pressure and 547 data points for diastolic blood pressure shows that blood pressure in childhood persists in later life [6]. The blood pressure tracking was greater for systolic than for diastolic blood pressure [6]. Second, our association between residential green space indicators early in life and adult blood pressure might be mediated or partly mediated by air pollution

It might be possible that low air pollution concentrations in areas with more surrounding greenness result in a lower blood pressure. Studies in adults show that a long-term exposure to air pollution is associated with higher blood pressure [3, 22–26]. The underlying pathway between particulate air pollution and blood pressure may be oxidative stress and inflammation which can affect placental function and fetal growth [27]. Third, besides air pollution, green space reduce exposure to noise. Noise may play a role in the association between residential greenness and blood pressure since noise exposure can result in a higher blood pressure [28, 29]. However, the models were robust for mutually adjustment for noise exposure during the night.

Studies on early-life stressors, such as ambient air pollution, noise and stress, that may influence blood pressure in adulthood are limited. One study shows that prenatal exposure to ambient air pollutants are associated with an increased risk for adverse cardiovascular health outcomes [30]. Another study in adults noted a negative association between prenatal life stress and blood pressure in young adulthood [31]. To our knowledge no studies have investigated prenatal noise exposure in association with adult blood pressure. Besides these early-life stressors, other factors including smoking behaviour are associated with adult blood pressure. With this regard, maternal smoking during pregnancy has been associated with an increase in offspring blood pressure in adulthood [32, 33].

A major advantage of this study is that we individually estimated greenness exposure of each participant. Our study is the first to include information on greenness exposure in the early-life when investigating adult blood pressure. Since we cannot differentiate between prenatal and postnatal exposure to residential greenness, our study is limited to define the exact time period of the exposure. Nevertheless, residential greenness in early-life remained significant even after additional adjustment for residential greenness exposure in adulthood. We found no significant effect of adult residential greenness with adult blood pressure, while accounting for the early-life greenness exposure.

Strengths include the use of ambulatory blood pressure and the possibility to control for potential confounding factors and covariates of blood pressure such as age, gender, smoking status, physical activity, BMI, sodium and potassium intake and alcohol consumption. Ambulatory blood pressure was measured at home during usual daily activities and is, compared to office measurements, a better predictor of all cause and cardiovascular mortality and has a moderate-to-relatively high tracking stability and predictability over time [34, 35]. There are a number of limitations to our study. The twins were born from 1975 till 1982, although during the last 30 years traffic volume did increase no major geographical changes did appear since no major changes in the road network have occurred in East-Flanders since 1974. In addition, we used land-cover data from 2000 as no earlier satellite data were available. However strong correlations have been shown over time between 2000 and 2006 [36]. A major limitation of our study is that information on residential location is only available for the first and last residence. This results in more accurate exposure estimations for non-movers than for the movers because no data is available on moment of moving and frequency of moving.

Since high blood pressure is the leading global risk factor for cardiovascular disease and mortality in the world [1], these findings have public health implications. Previous studies have shown that a small reduction in blood pressure at the population level could have a major impact in reducing morbidity and mortality [37]. Even, a 2 mmHg lower systolic blood pressure at the population level would result in an 10% overall reduction in mortality due to stroke mortality and about 7% lower mortality from ischemic heart disease or other vascular causes in middle age [37].

## Conclusions

Our findings indicate that more surrounding greenness at the residential address in early-life shows lower blood pressure in adult life. This may be especially relevant for policy makers and urban planners for designing healthier urban environments.

## Additional file

Additional file 1: Table S1. Pearson correlation coefficient between greenness in early-life and adult life. **Table S2.** Collinearity diagnosis. **Table S3.** Estimated difference in night systolic blood pressure (mm Hg) with land-use indicators at birth in all buffer sizes in twins who moved since birth. **Figure S1.** Ambulatory blood pressure (mm Hg) in association with residential greenness exposure with no adjustment for exposure in adulthood/early-life in twins who moved during life. **Figure S2.** Ambulatory blood pressure (mm Hg) in association with residential area. **Figure S3.** Ambulatory blood pressure (mm Hg) in association with industrial area. (DOCX 872 kb)
