# Supplementary material for: Blood pressure in young adulthood and residential greenness in the early-life environment of twins
Source: Environ Health. 2017 Jun 5;16:53. doi: 10.1186/s12940-017-0266-9 (PMC5460325; doi:10.1186/s12940-017-0266-9)
Supplement: Additional file 1: Table S1. — Pearson correlation coefficient between greenness in early-life and adult life. Table S2. Collinearity diagnosis. Table S3. Estimated difference in night systolic blood pressure (mm Hg) with land-use indicators at birth in all buffer sizes in twins who moved since birth. Figure S1. Ambulatory blood pressure (mm Hg) in association with residential greenness exposure with no adjustment for exposure in adulthood/early-life in twins who moved during life. Figure S2. Ambulatory blood pressure (mm Hg) in association with residential area. Figure S3. Ambulatory blood pressure (mm Hg) in association with industrial area. (DOCX 872 kb) [file 12940_2017_266_MOESM1_ESM.docx]

**Supplemental Material**

**Blood pressure in young adulthood and residential exposure of greenness in the early-life environment of twins**

**Esmée M Bijnens, PhD; Tim S Nawrot, PhD; Ruth JF Loos, MD, PhD; Marij Gielen, MD, PhD; Robert Vlietinck, MD, PhD; Catherine Derom, PhD; Maurice P Zeegers, PhD**

**Table of content**

| Table S1 | P 3. |
| --- | --- |
| Table S2 | P 3. |
| Table S3 | P 4. |
| Figure S1 | P 5. |
| Figure S2 | P 6 |
| Figure S3 | P 7 |

| Table 1: Pearson correlation coefficient between greenness in early-life and adult life | | | | | | | | | | |
| --- | --- | --- | --- | --- | --- | --- | --- | --- | --- | --- |
|  |  |  | | | | | | | |  |
|  |  | Residential greenness in early-life | | | | | | | |  |
|  |  | 5000 m | 4000 m | 3000 m | 2000 m | 1000 m | 500 m | 300 m | 100 m |  |
|  |  |  |  |  |  |  |  |  |  |  |
| Residential greenness in adult life | 5000 m | r=0.71*** | r=0.70*** | r=0.67*** | r=0.59*** | r=0.40*** | r=0.27** | r=0.23* | r=0.19 |  |
|  | 4000 m | r=0.70*** | r=0.70*** | r=0.68*** | r=0.61*** | r=0.43*** | r=0.28** | r=0.25* | r=0.21* |  |
|  | 3000 m | r=0.67*** | r=0.68*** | r=0.68*** | r=0.63*** | r=0.44*** | r=0.30*** | r=0.27** | r=0.22* |  |
|  | 2000 m | r=0.61*** | r=0.62*** | r=0.63*** | r=0.60*** | r=0.45*** | r=0.32*** | r=0.30*** | r=0.24* |  |
|  | 1000 m | r=0.40*** | r=0.40*** | r=0.42*** | r=0.41*** | r=0.32*** | r=0.26** | r=0.24* | r=0.21* |  |
|  | 500 m | r=0.24* | r=0.24* | r=0.26** | r=0.25* | r=0.18 | r=0.15 | r=0.13 | r=0.12 |  |
|  | 300 m | r=0.16 | r=0.17 | r=0.17 | r=0.16 | r=0.08 | r=0.06 | r=0.05 | r=0.05 |  |
|  | 100 m | r=0.16 | r=0.16 | r=0.16 | r=0.14 | r=0.06 | r=0.03 | r=-0.01 | r=0.02 |  |
|  | | | | | | | | | | |
| Asterix denote (*) P< 0.01, (**) P< 0.001, and (***) P<0.0001. | | | | | | | | | | |

| Table S2: Collinearity diagnosis | | | | | | | | |
| --- | --- | --- | --- | --- | --- | --- | --- | --- |
|  |  | | | | | | | |
|  | Variance Inflation Factor (VIF) | | | | | | | |
| Residential greenness | 5000 | 4000 | 3000 | 2000 | 1000 | 500 | 300 | 100 |
| Early-life | 2.70 | 2.62 | 2.47 | 2.06 | 1.38 | 1.28 | 1.26 | 1.19 |
| Adult life | 2.56 | 2.54 | 2.44 | 2.02 | 1.39 | 1.22 | 1.20 | 1.19 |

| Table S3 Estimated difference in night systolic blood pressure (mm Hg) with land-use indicators at birth in all buffer sizes in twins who moved since birth | | | | | | | | | |
| --- | --- | --- | --- | --- | --- | --- | --- | --- | --- |
|  | | **Nighttime systolic blood pressure** | | | | | | | |
|  | | **Beta** | | **95% CI** | | ***P*-value** | |  |  |
|  |  | | | | | | | | |
| *Semi-natural, forested and agricultural area (increase in IQR):* | | | | | | | | | |
| 5000 m, + 22% | | | -2.45 | | -4.7 to -0.2 | | 0.04 | |  |
| 4000 m, + 23% | | | -2.52 | | -4.6 to -0.4 | | 0.02 | |  |
| 3000 m, + 32% | | | -3.64 | | -6.2 to -1.1 | | 0.006 | |  |
| 2000 m, + 40% | | | -4.08 | | -6.9 to -1.3 | | 0.006 | |  |
| 1000 m, + 47% | | | -3.67 | | -6.4 to -0.9 | | 0.012 | |  |
| 500 m, + 47% | | | -2.87 | | -5.6 to -0.1 | | 0.043 | |  |
| 300 m, + 43% | | | -2.57 | | -5.1 to -0.1 | | 0.049 | |  |
| 100 m, + 29% | | | -1.51 | | -3.1 to 0.1 | | 0.07 | |  |
|  | | | | | | | | | |
| Adjusted for sex, gestational age, birth weight, birth year, zygosity–chorionicity group, maternal age, age, smoking, physical activity, BMI, 24h sodium and potassium, gamma-glutamyl transferase, indicators of socioeconomic status (maternal education and neighborhood household income), smoking during pregnancy and noise exposure during the night. We additionally adjust for greenness exposure in adulthood. | | | | | | | | | |

|  |  |  |
| --- | --- | --- |


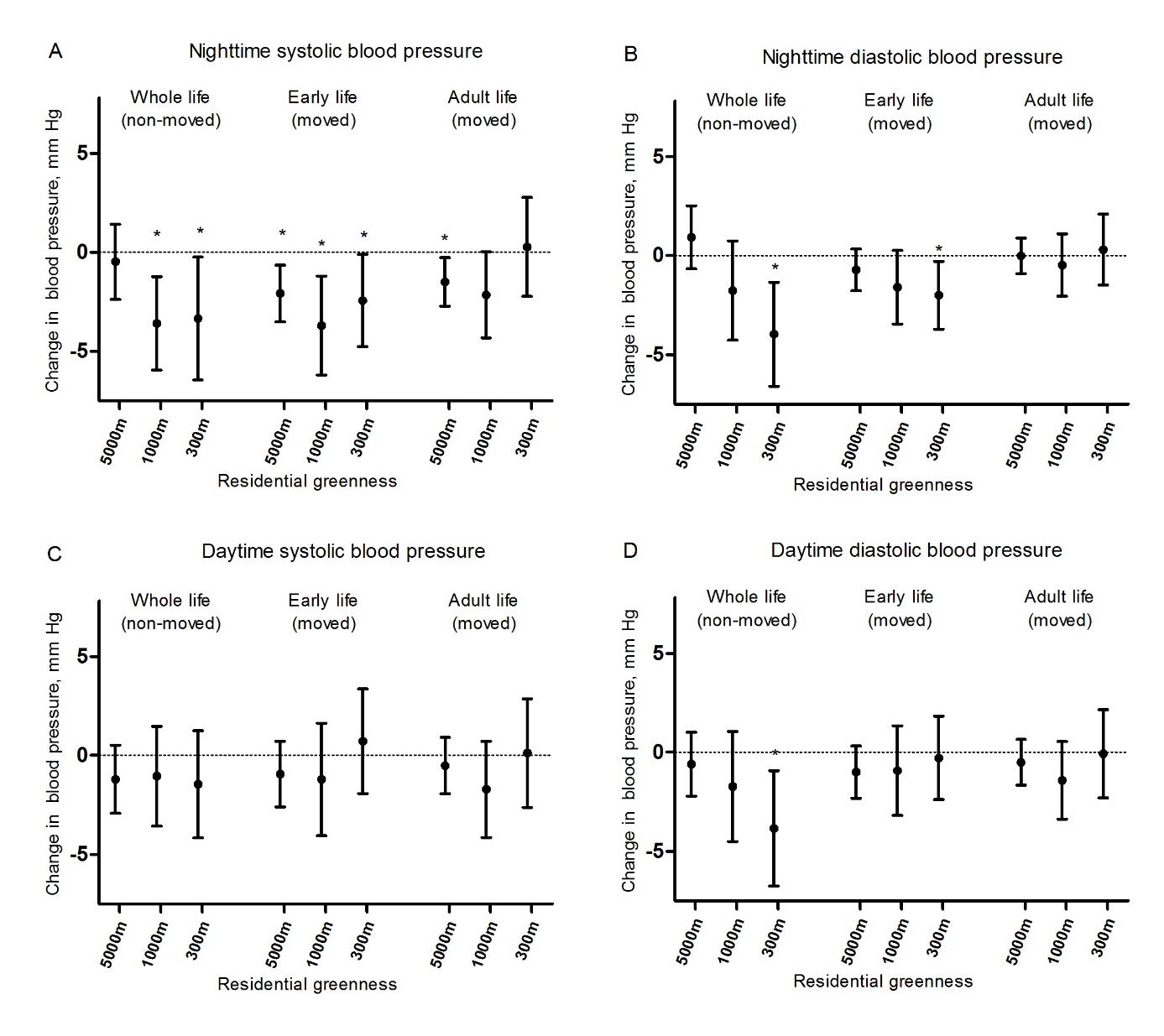
 **Figure S1: Ambulatory blood pressure (mm Hg) in association with residential greenness exposure with no adjustment for exposure in adulthood/early-life in twins who moved during life.** A) Night systolic, B) Night diastolic, C) Day systolic and D) Day diastolic blood pressure. Adjusted for sex, gestational age, birth weight, birth year, zygosity–chorionicity group, maternal age, age, smoking, physical activity, BMI, 24h sodium and potassium, gamma-glutamyl transferase, indicators of socioeconomic status (maternal education and neighborhood household income), smoking during pregnancy and noise exposure during the night. Vertical lines denote 95% confidence intervals. *indicates significant (p < 0.05) change in blood pressure for an IQR increase in land use indicators.

**
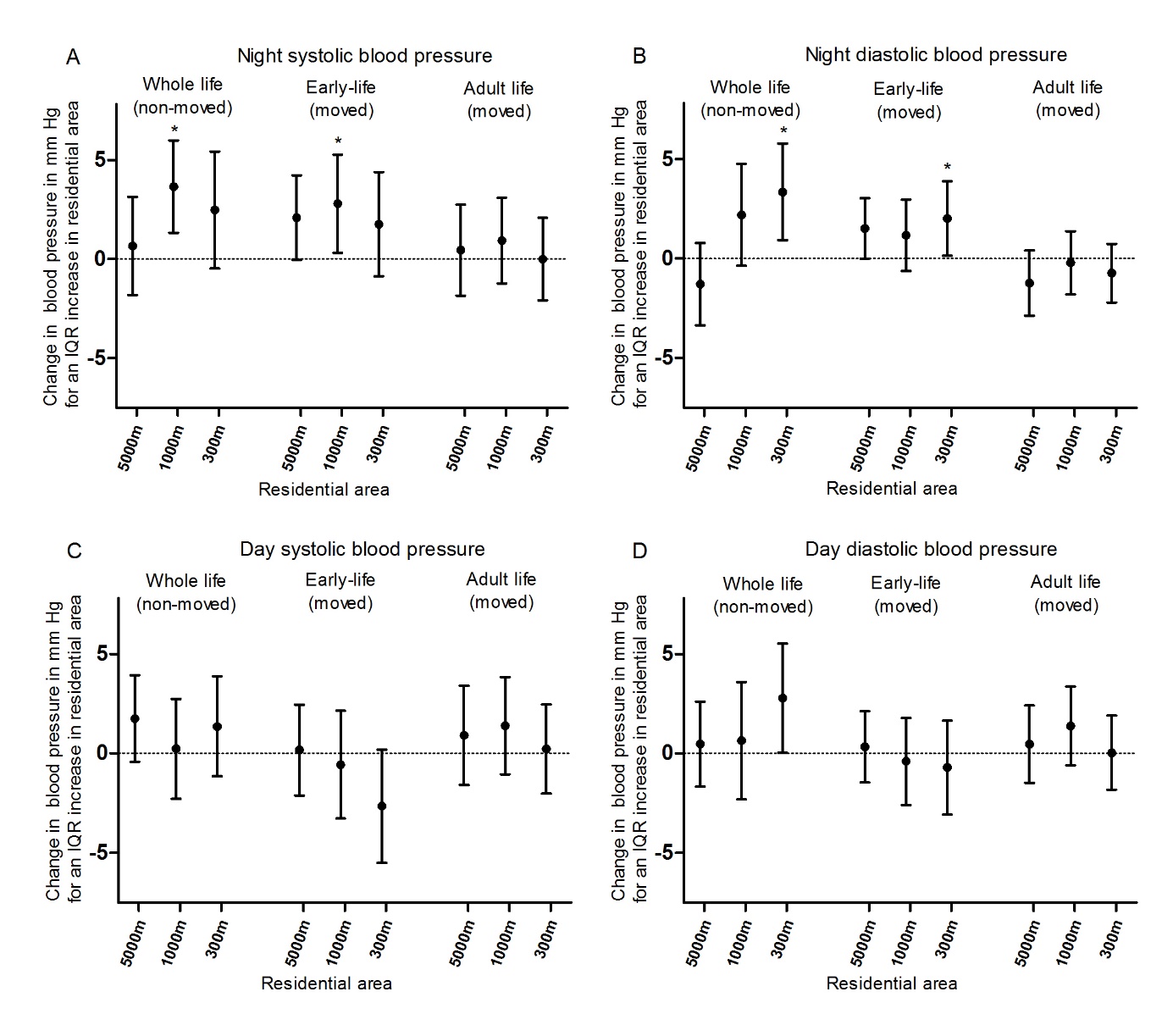
Figure S2: Ambulatory blood pressure (mm Hg) in association with residential area.** A) Night systolic, B) Night diastolic, C) Day systolic and D) Day diastolic blood pressure. Adjusted for sex, gestational age, birth weight, birth year, zygosity–chorionicity group, maternal age, age, smoking, physical activity, BMI, 24h sodium and potassium, gamma-glutamyl transferase, indicators of socioeconomic status (maternal education and neighborhood household income), smoking during pregnancy and noise exposure during the night. In the twins who moved during life, we additionally adjust for residential area in adulthood/early-life. Vertical lines denote 95% confidence intervals. *indicates significant (p < 0.05) change in blood pressure for an IQR increase in land use indicators.

**
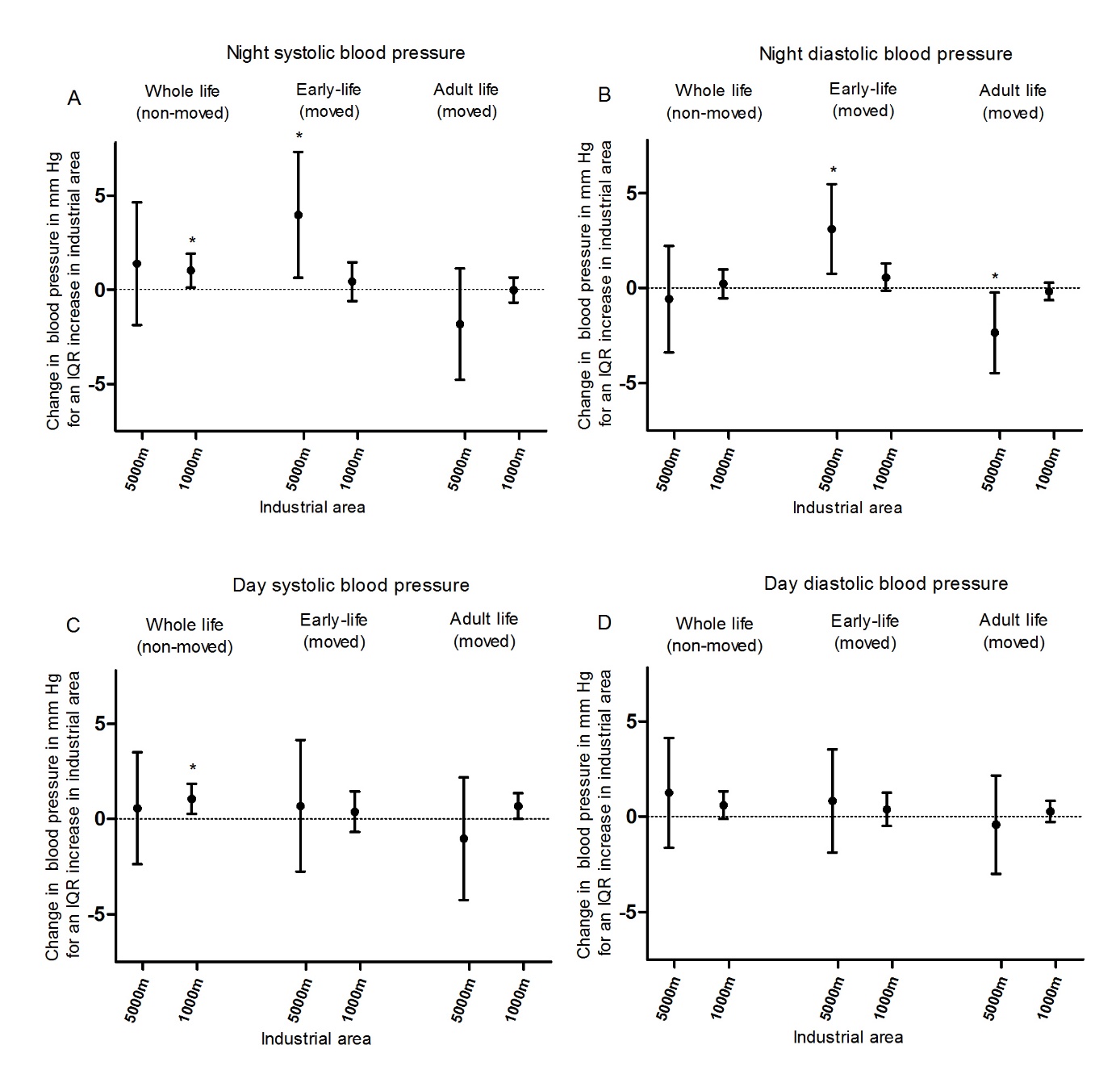
Figure S3: Ambulatory blood pressure (mm Hg) in association with industrial area .** A) Night systolic, B) Night diastolic, C) Day systolic and D) Day diastolic blood pressure. Adjusted for sex, gestational age, birth weight, birth year, zygosity–chorionicity group, maternal age, age, smoking, physical activity, BMI, 24h sodium and potassium, gamma-glutamyl transferase, indicators of socioeconomic status (maternal education and neighborhood household income), smoking during pregnancy and noise exposure during the night. In the twins who moved during life, we additionally adjust for industrial area in adulthood/early-life. Vertical lines denote 95% confidence intervals. *indicates significant (p < 0.05) change in blood pressure for an IQR increase in land use indicators.
